# A Common *CYFIP1* Variant at the 15q11.2 Disease Locus Is Associated with Structural Variation at the Language-Related Left Supramarginal Gyrus

**DOI:** 10.1371/journal.pone.0158036

**Published:** 2016-06-28

**Authors:** Young Jae Woo, Tao Wang, Tulio Guadalupe, Rebecca A. Nebel, Arianna Vino, Victor A. Del Bene, Sophie Molholm, Lars A. Ross, Marcel P. Zwiers, Simon E. Fisher, John J. Foxe, Brett S. Abrahams

**Affiliations:** 1 Department of Genetics, Albert Einstein College of Medicine, Bronx, United States of America; 2 Department of Epidemiology & Population Health, Albert Einstein College of Medicine, Bronx, United States of America; 3 Language and Genetics Department, Max Planck Institute for Psycholinguistics, Nijmegen, the Netherlands; 4 The Sheryl and Daniel R. Tishman Cognitive Neurophysiology Laboratory, Children's Evaluation and Rehabilitation Center (CERC), Albert Einstein College of Medicine, Bronx, United States of America; 5 Department of Pediatrics, Albert Einstein College of Medicine, Bronx, United States of America; 6 Donders Institute for Brain, Cognition and Behaviour, Radboud University, Nijmegen, The Netherlands; 7 The Cognitive Neurophysiology Laboratory, Nathan S. Kline Institute for Psychiatric Research, Orangeburg, United States of America; 8 Dominick P. Purpura Department of Neuroscience, Albert Einstein College of Medicine, Bronx, United States of America; Benito Menni Complejo Asistencial en Salud Mental, SPAIN

## Abstract

Copy number variants (CNVs) at the Breakpoint 1 to Breakpoint 2 region at 15q11.2 (BP1-2) are associated with language-related difficulties and increased risk for developmental disorders in which language is compromised. Towards underlying mechanisms, we investigated relationships between single nucleotide polymorphisms (SNPs) across the region and quantitative measures of human brain structure obtained by magnetic resonance imaging of healthy subjects. We report an association between rs4778298, a common variant at *CYFIP1*, and inter-individual variation in surface area across the left supramarginal gyrus (lh.SMG), a cortical structure implicated in speech and language in independent discovery (n = 100) and validation cohorts (n = 2621). *In silico* analyses determined that this same variant, and others nearby, is also associated with differences in levels of *CYFIP1* mRNA in human brain. One of these nearby polymorphisms is predicted to disrupt a consensus binding site for FOXP2, a transcription factor implicated in speech and language. Consistent with a model where FOXP2 regulates *CYFIP1* levels and in turn influences lh.SMG surface area, analysis of publically available expression data identified a relationship between expression of FOXP2 and *CYFIP1* mRNA in human brain. We propose that altered *CYFIP1* dosage, through aberrant patterning of the lh.SMG, may contribute to language-related difficulties associated with BP1-2 CNVs. More generally, this approach may be useful in clarifying the contribution of individual genes at CNV risk loci.

## Introduction

Rare multi-gene copy number variants (CNVs) are well established to increase risk for neurodevelopmental disorders, but translational efforts have been hindered by a limited understanding of underlying mechanisms. The four gene region between breakpoints 1 and 2 (BP1-2) at 15q11.2 is interesting in this regard in that deletions are associated with increased risk for epilepsy and schizophrenia [1–6], and the reciprocal duplications may be relevant to autism [7–9]. Language appears to be compromised in a large number of CNV carriers, in that a meta-analysis of clinically ascertained BP1-2 CNV carriers determined that speech delay was present in 92% and 49% of deletion and duplication subjects, respectively (8). Similarly, separate work found evidence for a significant association between deletion status and self-reported reading difficulties (e.g. “Did you experience any difficulties in reading in elementary school?”) [4]. Described below is work aimed at providing molecular insights into how genetic variation at the BP1-2 region may contribute to disease-related variation in human brain structure.

Recent work found a relationship between gene dosage at BP1-2 and structural variation at multiple disease associated brain regions [4]. Through analysis of magnetic resonance imaging (MRI) data from BP1-2 deletion, duplication, and CNV neutral subjects, investigators identified linear relationships between gene dosage and volume of the left insula (lh.insula), right anterior cingulate cortex (rh.ACC), corpus callosum (CC), left temporal white matter (lh.tempWM), and the left supramarginal gyrus (lh.SMG). The volumes of the lh.insula, rh.ACC, lh.SMG, and lh.tempWM were reduced in deletion carriers and increased in duplication carriers, whereas the opposite directionality was observed for the CC. Interestingly, people with schizophrenia are reported to show reduced volumes of the insula, CC, and lh.tempWM [10–12], as well as abnormal connectivity of the rh.ACC [13]. Structural and functional alterations of the lh.SMG have been implicated in speech and language [14,15]. Taken together, these data support a model whereby altered gene dosage at BP1-2 influences regional brain development and increases risk for disease. We hypothesized that common regulatory variants at the locus might be associated with similar effects in healthy individuals, and that their identification might clarify mechanisms of disease.

The BP1-2 region at 15q11.2 contains four genes (*TUBGCP5*, *CYFIP1*, *NIPA2*, and *NIPA1*); each has interesting connections to neurodevelopment. *TUBGCP5* encodes a protein involved in regulation of chromosomal segregation [16–18]. Although not yet studied with regard to disease, it is directly regulated by GSK3beta, implicated in schizophrenia [19–21]. More is known about the adjacent gene that encodes CYFIP1. Binding to RAC1 activates the Wave Regulatory Complex (WRC) and initiates cytoskeletal remodeling [22,23]. A separate interaction between CYFIP1 and the fragile X mental retardation protein (FMRP) results in the repression of eIF4E-mediated translation [24,25]. Knockdown or overexpression of *CYFIP1* impacts neuronal morphology, brain development and function [26–29] and common *CYFIP1* regulatory variants have been associated with schizophrenia and autism [2,28,30]. Separate studies looking at *NIPA2*, a third gene in the region, identified rare functional variants in epilepsy patients but not controls [31,32] and found a schizophrenia associated single nucleotide polymorphism (SNP) upstream of the gene [2]. Moreover, investigation of the relationship between regional gene expression and behavior in individuals with Prader-Willi Syndrome found strong correlations with *NIPA2* [33]. *NIPA1*, immediately downstream of *NIPA2*, has been implicated in axonal growth [34]. Related, is that dominant negative mutations in *NIPA1* cause hereditary spastic paraplegia [35].

In this study, examination of a discovery cohort identified SNPs associated with structural variation at brain regions known to be sensitive to gene dosage at the BP1-2 region. The top hit, upstream of *CYFIP1*, was likewise associated with variation in lh.SMG surface area in a large independent cohort. Consistent with a gene dosage effect, interrogation of publically available expression data revealed a relationship between genotype at this variant and *CYFIP1* levels in human brain. Additional *in silico* analyses we performed suggest that this association with *CYFIP1* expression may be mediated by allele-dependent regulation by FOXP2, a transcription factor (TF) implicated in speech and language [36–38]. Results provide independent support for previous work identifying a relationship between genetic variation at BP1-2 and human brain structure, and point to *CYFIP1* as a likely contributor. The novel link to FOXP2 provides convergent evidence for involvement of the BP1-2 region in language. The approach used here could be useful in understanding risk mechanisms at other disease-related CNVs.

## Materials and Methods

### Discovery Cohort: Subjects, MRI, and Genotyping

The Einstein Institutional Review Board approved this study and subject recruitment. All participants gave informed consent prior to participation. 100 unrelated adults without any self-reported physical disability, psychiatric diagnosis, or first degree relatives with a psychiatric diagnosis were recruited for MRI imaging (S1 Table). 81 of these individuals were Caucasian. Saliva was collected using Oragene OG-500 kits (DNA Genotek). T1-weighed anatomical images were acquired using a Philips 3T Achieva Quasar TX scanner with 32 channel SENSE head coils. An MPRAGE protocol with the following parameters was used: TR/TE = 8.187/3.724 ms, TFE prepulse inversion time = 900ms, flip angle = 8°, 220 axial slices (1.0 mm thick) with 256x256 acquisition matrix. 101 HapMap SNPs with a minor allele frequency (MAF) ≥15% mapped to the BP1-2 interval (chr15:22783636–23085559; Hg19). Tagger was used to define 57 tag-SNPs (r^2^≥0.8, CEU/TSI populations from Hapmap Version 3, Release 2; S1 Fig and S2 Table) [39]. DNA was extracted using QuickGene SP kits (Kurabo) and a QuickGene 610 instrument (Autogen). Integrity was verified via electrophoresis and concentration measured using a NanoDrop 2000 (Thermo). Primers for Sequenom genotyping were designed for 56/57 SNPs that could be multiplexed [40]. Genotype quality was evaluated by visual examination of clustering, testing for Hardy-Weinberg equilibrium, and retyping of random subjects. 52 SNPs remained after quality control.

### Validation Cohort—Subjects, MRI, and Genotyping

Subjects were healthy adults of Dutch descent included in the Nijmegen Brain Imaging Genetics cohort [41,42]. None reported any neurological or psychiatric history. Research was approved by the CMO Region Arnhem-Nijmegen ethics committee. All participants gave written informed consent prior to participation. Imaging data was available or generated for 1276 subjects (all Caucasian) typed for each of rs12102024 and rs1051288, and 2621 individuals (2383 Caucasian) typed for rs4778298 (S3 Table). T1-weighted anatomical scans were acquired for previous work using a 1.5T or 3T Siemens scanner. rs12102024 and rs1051288 genotypes, obtained by Affymetrix GeneChip SNP 6.0 array, were likewise generated previously [43]. rs4778298 was genotyped for this study at the Max Planck Institute in Nijmegen using a custom KASP assay (LGC Genomics). The assay was validated by Sanger sequencing of randomly-selected samples.

### Image processing

Scans were processed using FreeSurfer 5.3 [44,45]. For discovery cohort subjects, segmentation was confirmed by inspection of a midline saggital section. Volumes, surface areas, and thicknesses for all regions in the Desikan-Killany atlas [46] were acquired. Analyses were, however, tightly restricted to the lh.insula, rh.ACC, CC, lh.temp.WM, and lh.SMG, regions of interest (ROIs) shown previously to be sensitive to CNVs at BP1-2 [4]. Volume of the rh.caudalanteriorcingulate was taken to represent the rh.ACC, and the volumes of five individually segmented callosal subregions (anterior, mid anterior, central, mid posterior, and posterior) as the CC. lh.tempWM was defined as white matter within the: left bank of the superior temporal sulcus, left entorhinal cortex, left fusiform gyrus, left inferior-temporal cortex, left middle-temporal cortex, left parahippocampal cortex, left superior-temporal cortex, and left temporal pole. Because FreeSurfer does not output surface area or thickness of subcortical regions, the CC and lh.temp.WM were only included for analysis of volume.

### Statistical Analyses

In the discovery cohort, associations of individual SNP-ROI pairs were examined using R [47] according to the following linear regression model: Volume of ROI = α + β1(variant) + β2(age) + β3(gender) + β4(ethnicity) + β5(handedness) + β6(total brain volume) + ε, in which α is the intercept and ε random error. For analysis of surface area and cortical thickness, total brain surface area and mean cortical thickness of the whole brain were used for β6, respectively. To assess the aggregate effect of individual SNPs across ROIs, the sum of chi-square statistics (X^2^_0_) was calculated and its significance (p_permutation corrected_ < 0.05) was evaluated by permutation with 10,000 replicates (X^2^_n_). By aggregating effects across ROIs for each SNP, we were able to perform only one test for each anatomical trait (volume, surface area, and thickness), improving power to detect any effects present. In the validation cohort, three SNP-ROI pairs were tested in PLINK [48] under a linear regression model as above, but scanner field strength was included and handedness omitted. Handedness was not included as a covariate in our analysis of data from the validation cohort. Left handed individuals made up less than 5% of subjects and previously published work in this cohort saw no relationship between handedness and any regional measure of surface area [49].

### Gene Expression

Normalized expression data (Human Exon 1.0 ST, Affymetrix) for 1231 human brain samples from 134 individuals (16–102 at death; mean age 59) and corresponding genotypes (Omni 1M Immunochip, Illumina) were downloaded from BRAINEAC [50]. The relationship between expression (averaged across brain regions) and genotype was modeled using linear regression to identify expression quantitative trait loci (eQTL) variants. No correction for multiple comparisons was performed because only one probe (Affymetrix 3583676), specific for the 5’ UTR of *CYFIP1*^short^, was tested. For evaluating the relationship between levels of *CYFIP1*^short^ and candidate TFs, Pearson's correlation coefficients were calculated, and significance assessed using an F-test based on a lack-of-fit sum-of-squares method.

### TF Binding

50 bps of genomic sequence on either side of each *CYFIP1*^short^ eQTL variant was extracted from the UCSC browser (Hg19). Sequences (1 per allele) were screened against human Position Weight Matrices (PWMs) in JASPAR, a database of TF binding profiles, using the Transcription Factor Binding Site Tools package [51,52]. Matches were defined as having a relative score ≥0.8. For differential binding, information content ≥1 at the variable base within the PWM was required.

## Results

### Common genetic variation at BP1-2 is associated with inter-individual differences in brain structure

**Fig 1:**
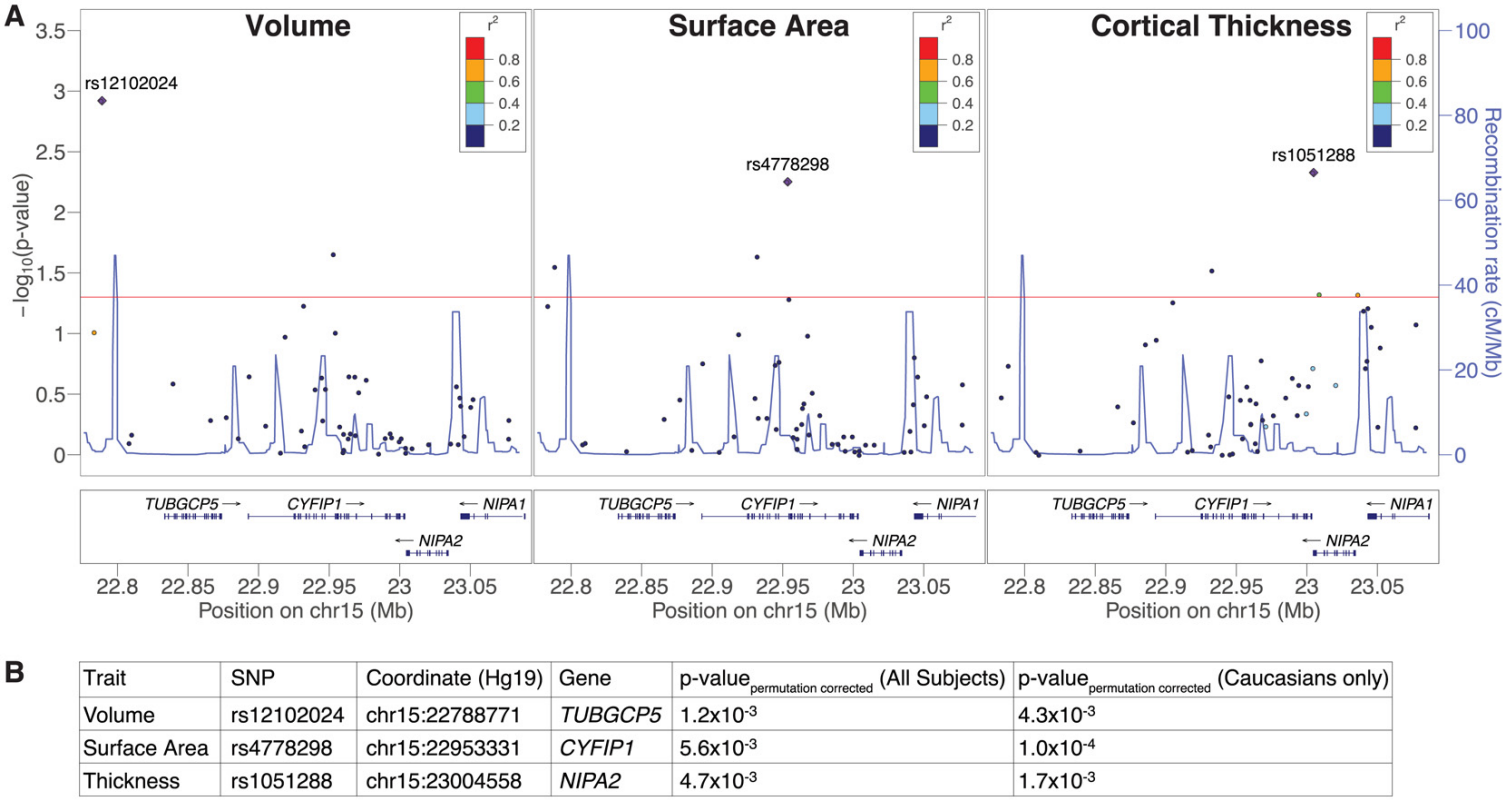
Common variant genotypes at 15q11.2 arssociated with differences in regional cortical volume, thickness, and surface area in a discovery cohort. (A) Aggregate -log p-values for single nucleotide polymorphisms (SNPs) are plotted against genomic position. P-values reflect the relationships between genotype in the discovery cohort and volume (left), surface area (middle), or thickness (right) across all regions of interest (ROIs). Significant associations (p_permutation corrected_<0.05) appear above the horizontal red line. Linkage disequilibrium of top hits (diamonds) to typed variants is specified, and estimated recombination rates plotted (blue lines). r^2^ in the legend indicates the correlation between genotype at each tag-SNP (circles) and genotype at the top hit (diamond). (B) Top aggregate p-values and their SNP descriptions for each trait examined in the full cohort and the Caucasian subset are summarized.

**Fig 2:**
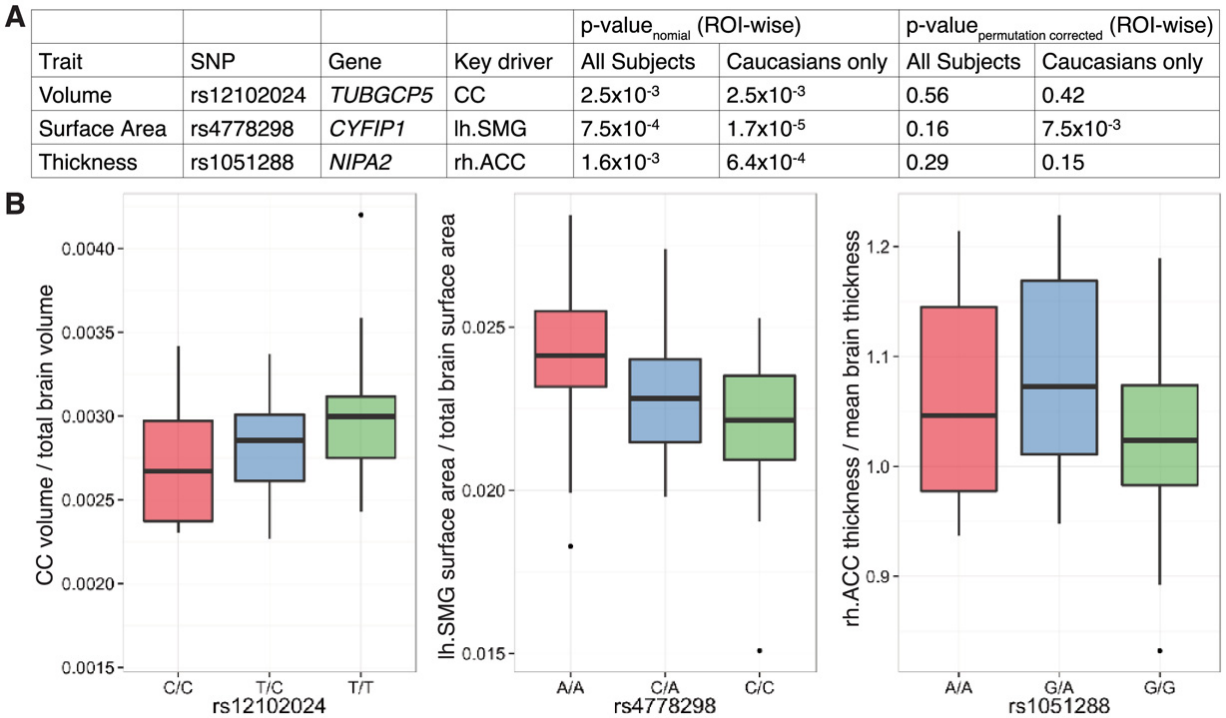
Individual regions of interest (ROIs) drive associations observed in the discovery cohort. (A) Pairwise analyses of the top signal for each of volume, surface area, and thickness from the discovery cohort determined that aggregate effects were attributable to individual ROIs (and see S5 Table). Post-hoc permutation testing, accounting for all 52 tag-SNPs and ROIs examined, found a significant association between rs4778298 genotype and the surface area of the left supramarginal gyrus (lh.SMG) in Caucasian subjects (p_permutation corrected_ = 7.5x10^-3^). (B) Linear effects at the CC and lh.SMG were observed for rs12102024 and rs4778298, respectively, consistent with an effect of allele dosage. rh.ACC and CC correspond to right anterior cingulate cortex and corpus callosum, respectively.

We performed targeted genetic association analyses in a discovery cohort (S1 Table). 52 tag-SNPs from the BP1-2 region were genotyped (S2 Table), and the relationships to volume, surface area, and thickness at predefined ROIs examined under an additive model. Hypothesizing that common regulatory variants within the interval would give rise to effects like those seen in rare CNV carriers, we restricted analyses to ROIs shown previously to be sensitive to BP1-2 dosage [4]. Significant associations were observed (Fig 1A and S4 Table). The strongest signal (rs4778298 at *CYFIP1*; p_permutation corrected_ = 5.6x10^-3^; Fig 1B) became more pronounced after limiting analysis to Caucasians (p_permutation corrected_ = 1.0x10^-4^; Fig 1B). Finally, even if an overly stringent Bonferonni correction is used to account for the 3 endpoints examined, p-values of 1.7x10^-2^ and 3.3x10^-4^ are observed. Counter to our initial hypothesis that common regulatory variants might uniformly impact all BP1-2 sensitive brain regions, effects were attributable to individual ROIs (Fig 2A and S5 Table). A post-hoc permutation analysis, accounting for all 52 tag-SNPs and ROIs, revealed a significant association between rs4778298 and lh.SMG surface area in Caucasians (p_permutation corrected_ = 7.5x10^-3^; see S6 Table). Linear effects at the CC and lh.SMG were observed for rs12102024 and rs4778298, respectively, consistent with an effect of gene dosage (Fig 2B).

### Relationship between SNP genotype at *CYFIP1* and left supramarginal gyrus surface area in an independent population

**Fig 3:**
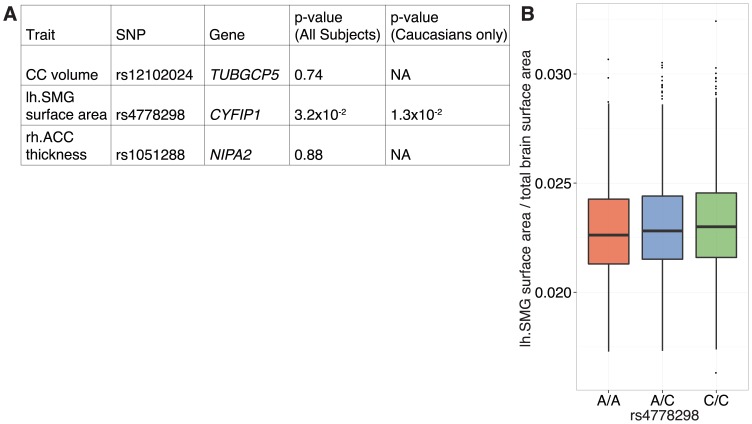
Linear association between common variant genotype at *CYFIP1* and surface area across the left supramarginal gyrus (lh.SMG) in an independent cohort. (A) An association between rs4778298 genotype and lh.SMG surface area was observed (p_nominal_ = 3.2x10^-2^) in a large independent population (n = 1276 for rs12102024 and rs1051288; n = 2621 for rs4778298). Similar to what we observed in our discovery cohort, this effect became stronger on analysis of Caucasian subjects alone and survived correction for multiple comparisons (p_nominal_ = 1.3x10^-2^; p_bonferroni corrected_ = 4.9x10^-2^). (B) As in the discovery cohort, a linear relationship between genotype and lh.SMG surface area was observed. Notably, however, directionality was the reverse of what was seen previously. rh.ACC and CC correspond to right anterior cingulate cortex and corpus callosum, respectively.

To assess the generalizability of our findings, we analyzed the top three hits in an independent cohort of healthy adults [43]. Imaging data was available for 1276 subjects typed for each of rs12102024 and rs1051288, and 2621 individuals typed for rs4778298 (S3 Table). We saw no signal at rs12102024 or rs1051288, but did see an effect at rs4778298, the top hit in our discovery cohort (p_nominal_ = 3.2x10^-2^; Fig 3). As above, the effect became stronger after restricting the analysis to Caucasians and survived correction for multiple comparisons (p_nominal_ = 1.3x10^-2^; p_bonferroni corrected_ = 4.9x10^-2^; Fig 3). A linear relationship between genotype and lh.SMG surface area was also observed as above, although in contrast to the discovery cohort the ‘A’ allele was seen to be associated with a relative reduction.

### Common variant signal associated with *CYFIP1* levels in human brain

**Fig 4:**
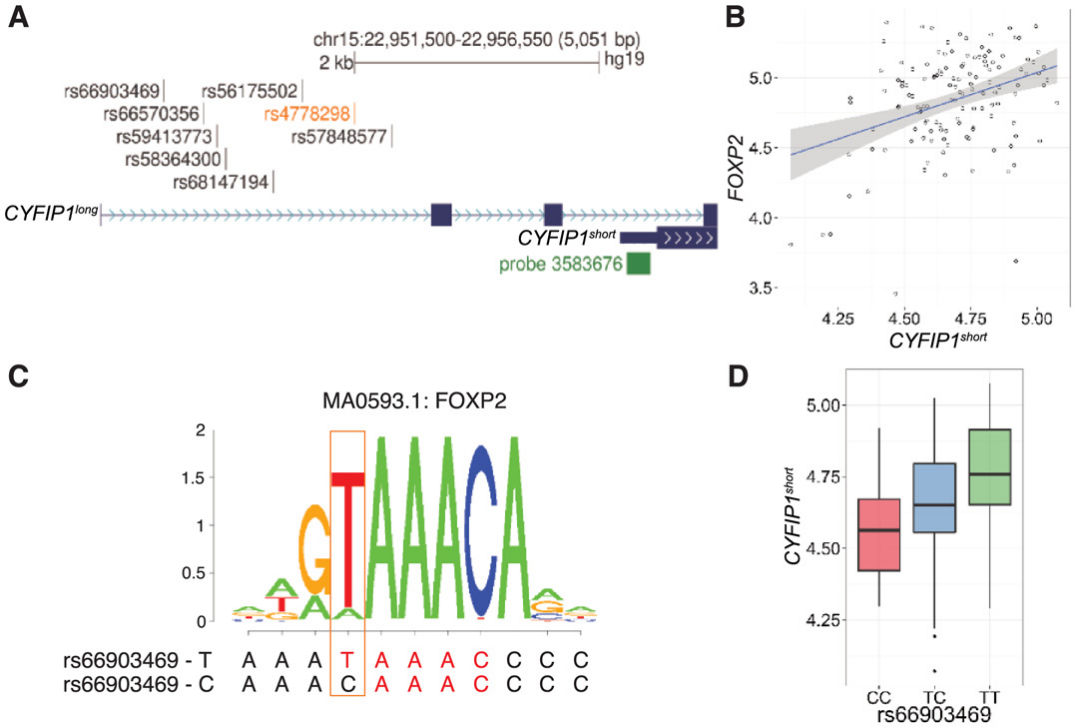
Evidence for genotype-dependent activation of *CYFIP1* by FOXP2. (A) rs4778298 (orange) associated with variation across the surface area of the left supramarginal gyrus is 2.2 kb upstream of *CYFIP1*^*short*^. Seven linked variants (r^2^>0.9), and an expression probe specific for *CYFIP1*^*short*^ (Affymetrix 3583676), are shown. (B) A significant positive association between levels of *FOXP2* and *CYFIP1* is seen in human brain (r^2^ = 0.36; p = 2.5x10^-5^). (C) rs66903469-T is predicted to bind FOXP2 with 11.8-fold greater affinity than the alternate C allele. MA0593.1 corresponds to the position weight matrix for FOXP2 defined in JASPAR. (D) rs66903469-T (r^2^ with rs4778298-C of 0.93), is strongly associated with increased levels of *CYFIP1*^*short*^ (p = 2.9x10^-5^).

Inspection of the genomic region surrounding rs4778298 determined that it was 2.2 kb upstream of the transcriptional start for a *CYFIP1* isoform encoding a 95 kDa protein (CYFIP1^short^; Fig 4A). To test the hypothesis that rs4778298 genotype is associated with variation in mRNA levels for *CYFIP1*^*short*^, we turned to BRAINEAC, a resource integrating genotype and expression data for human brain samples [50]. Analyses identified a significant linear relationship between rs4778298 genotype and *CYFIP1*^*short*^ levels (Affymetrix Probe 3583676; p = 7.2x10^-5^; S7 Table). rs4778298-C was associated with increased mRNA levels. Additional eQTL variants in high linkage disequilibrium (LD) with rs4778298 (r^2^>0.9; S7 Table) were also identified. No relationship between genotype at any of these SNPs and levels of *TUBGCP5*, *NIPA2*, or *NIPA1* was observed (p>0.05).

### Evidence for genotype-dependent activation of *CYFIP1* by FOXP2

We sought next to understand how genetic variation upstream of *CYFIP1*^*short*^ might impact expression and perhaps brain structure. Beginning with TFs in JASPAR for which binding in human was characterized (n = 127), we identified a subset of twenty-eight whose expression in brain was correlated with *CYFIP1*^short^ (p_bonferroni corrected_<0.05; S8 Table). Ten of these were predicted to bind in a genotype dependent manner to one or more of the eight *CYFIP1*^*short*^ eQTL variants we identified (S9 Table). After removing candidate TFs on the basis of logical inconsistencies (e.g. negatively correlated with *CYFIP1*^*short*^ expression but having a binding site containing the allele associated with increased expression), or where binding was unlikely to be sensitive to genotype (see methods; S9 Table), only E2F4 and FOXP2 remained. FOXP2 is particularly interesting, as it is well recognized as an important contributor to human speech and language [36–38]. We see a positive relationship between *FOXP2* and *CYFIP1*^*short*^ levels (r^2^ = 0.36; p = 2.5x10^-5^; Fig 4B). Consistent with these findings, the rs66903469-T allele predicted to bind FOXP2 with 11.8-fold greater affinity than the alternate C allele (Fig 4C) is associated with higher levels of *CYFIP1*^short^ (p = 2.9x10^-5^; Fig 4D and S9 Table).

## Discussion

Towards insight into how genetic variation at the 15q11.2 BP1-2 region may influence disease risk, we examined the relationship between tag-SNPs spanning the interval and aspects of regional brain structure. We report a common variant 2 kb upstream of *CYFIP1* associated with the surface area of the lh.SMG, a brain region implicated in language. A relationship between this genotype and lh.SMG surface area was also observed in an independent population composed of 2621 individuals. Consistent with a model whereby the effect is mediated by altered gene expression, *in silico* analyses revealed that genotype at this SNP, and at seven other variants in high LD, is associated with variation in *CYFIP1*^*short*^ mRNA levels in human brain. Differential expression may be mediated by FOXP2, a TF implicated in speech and language [36–38], as it is predicted to bind one of these eQTL variants in an allele-dependent fashion. Supportive of this idea, we report a correlation between mRNA levels for *FOXP2* and *CYFIP1*^*short*^ in human brain.

Although we see a relationship between rs4778298 genotype in two independent cohorts, the direction of the effect differs between the populations. The ‘A’ allele is associated with increased lh.SMG surface area in the discovery cohort, but a decrease in the validation cohort. While we cannot rule out the possibility that this result may be a false positive, the linearity of the observed effects that recapitulate those for rare CNVs at BP1-2 argue against this [4]. Moreover, as described below, the link to FOXP2 provides biological plausibility for the observed association. The difference in directionality we observed in the two cohorts we looked at may be the result of genetic interactions with variants at the BP1-2 locus or elsewhere in the genome [53,54]. Multiple *CYFIP1* regulatory variants independent from rs4778298 / rs66903469 have been associated with increased risk for schizophrenia and autism [2,28,30,55]. Further underscoring complexity at the locus, in one of these studies no association with schizophrenia was observed when the *CYFIP1* regulatory variant rs4778334 was considered in isolation, but a clear signal was seen when the effect of a second eQTL variant at the *ACTR2* gene encoding an interacting protein was taken into account [28]. Importantly, directionality was reversed as a function of this second genotype, with rs4778334-C seen to be protective in rs268864-AA individuals but found to be risk associated in rs268864-GG subjects. The difference in directionality we observed between the two populations we looked at could also be related to other variables. For example, parent of origin effects in abnormalities of synaptic transmission and behavior in *Cyfip1*^+/-^ have been reported [56]. Similarly, socioeconomic factors are known to be associated with the regional surface area of numerous brain regions including those involved in language [57]. The relatively small number of subjects within the discovery cohort may also be relevant. Although relatively little is known about CYFIP1 function in mammalian brain development, early knockdown of *Cyfip1* in mouse results in inappropriate outward migration of radial glia (RG) from the ventricular zone [28]. Our findings are consistent with this result, in that separate work in human suggests that RG are important regulators of cortical surface area [58,59]. Our results also provide potential insights into mechanisms that may mediate these effects. Full length CYFIP1 binds both RAC1 and FMRP, whereas CYFIP1^short^ is truncated and binds only FMRP [22,25]. All else being equal, less CYFIP1^short^ would result in fewer FMRP binding sites and increase competition between FMRP and RAC1 for CYFIP1^long^. Reduced RAC1 binding would be expected to dampen WRC activity and in turn interfere with RG migration, a hypothesis that could be tested experimentally. Such experiments, together with studies in additional cohorts, might also shed light on regional specificity. As it stands, it’s unclear whether the signal we observed at rs4778298 / rs66903469 is specific for the lh.SMG or alternatively a result of insufficient power. Results from these investigations will provide new insights into the relationship between genetic variation at the BP1-2 region and brain patterning.

The novel relationship we describe between *FOXP2* and *CYFIP1* is likewise intriguing given involvement of FOXP2 in the production and comprehension of speech and language. A point mutation disrupting the DNA-binding domain of FOXP2 was seen to segregate with a severe speech and language disorder in the multi-generational KE family [37,60], and screening of unrelated individuals with similar problems identified additional subjects harboring FOXP2 mutations [61]. Experimental work will be required to establish that FOXP2 binding at rs66903469 is genotype-dependent and that variation here is sufficient to alter *CYFIP1*^*short*^ levels, efforts that may be complicated by temporal and regional specificity. Nevertheless, the previously unknown relationship between *FOXP2* and *CYFIP1*^*short*^ provide new insight into language-related aspects of human brain development. Moreover, in keeping with our model that the lh.SMG may be sensitive to FOXP2 activity, an fMRI study determined that KE mutation carriers show hypo-activity at this region during a word retrieval task [62].

Although we did not evaluate directly the relationship between common variation at *CYFIP1* and language ability in this study, our results are consistent with the hypothesis that language impairment in BP1-2 deletion carriers [4,8] may be attributable to reduced *CYFIP1* acting on the lh.SMG. With regard to idiopathic disease, a meta-analysis points to a reduced volume of lh.SMG in dyslexic children prior to the onset of reading impairment [63,64]. Similarly, adults with dylexia show under-activation of the lh.SMG in a number of fMRI studies [65–67]. In terms of function, the lh.SMG as a part of the dorsal lexical pathway is known to be important in transforming written language components (graphemes) into phonological objects (phonemes) [68,69]. Another function of the lh.SMG is the integration of auditory and sensory inputs before forwarding signals to other brain regions relevant to speech articulation [70,71]. Based on these findings, evaluation of phonological processing and speech articulation in BP1-2 CNV carriers is warranted. Separate studies characterizing the relationships between common variation at the locus and language related outcomes in healthy individuals might complement these efforts.

Lastly, our study points to overlap between the effect of rare and common genetic variation at BP1-2 locus. Given practical difficulties assembling cohorts where all individuals harbor the same rare variant, and progress establishing large cohorts with both MRI and genetic data, the approach described here may be useful in understanding the mechanisms underlying risk for disease at other multi-gene CNVs.

## Supporting Information

S1 Fig Linkage disequilibrium (LD) structure of the Breakpoint 1 to Breakpoint 2 locus. Haploview was used to generate a schematic representation of the interval and define LD structure. Genes within the interval are illustrated (*TUBGCP5*, *CYFIP1*, *NIPA2*, *and NIPA1*). Below this, SNPs are represented by short vertical lines. LD blocks appear as black triangles.
(PDF)

S1 Table Description of Discovery Cohort. (XLSX)

S2 Table Genotyping primers used for multiplexing 15q11.2 disease region between BP1-2. (XLSX)

S3 Table Description of Validation Cohort. (XLSX)

S4 Table Permutation corrected p-values for all aggregate analyses in the Discovery Cohort. (XLSX)

S5 Table Nominal p-values for all pairwise analyses in the Discovery Cohort. (XLSX)

S6 Table Subject level rs4778298 genotypes and MRI data for Einstein Discovery Cohort. (XLSX)

S7 Table Relationship between *CYFIP1*^*short*^ expression and rs4778298 genotype (and seven variants in strong linkage dysequilibrium). (XLSX)

S8 Table Correlations between expression levels for candidate transcription factors and *CYFIP1*^*short*^. (XLSX)

S9 Table Prioritization of candidate transcription factors correlated with *CYFIP1*^*short*^. (XLSX)

## References

[1] Stefansson H, Rujescu D, Cichon S, Pietiläinen OPH, Ingason A, Steinberg S, et al Large recurrent microdeletions associated with schizophrenia. Nature. 2008;455: 232–6. 10.1038/nature07229 18668039 PMC2687075

[2] Zhao Q, Li T, Zhao X, Huang K, Wang T, Li Z, et al Rare CNVs and Tag SNPs at 15q11.2 Are Associated With Schizophrenia in the Han Chinese Population. Schizophr Bull. 2012; 10.1093/schbul/sbr197 PMC3627771 22317777

[3] De Kovel CGF, Trucks H, Helbig I, Mefford HC, Baker C, Leu C, et al Recurrent microdeletions at 15q11.2 and 16p13.11 predispose to idiopathic generalized epilepsies. Brain. 2010;133: 23–32. 10.1093/brain/awp262 19843651 PMC2801323

[4] Stefansson H, Meyer-Lindenberg A, Steinberg S, Magnusdottir B, Morgen K, Arnarsdottir S, et al CNVs conferring risk of autism or schizophrenia affect cognition in controls. Nature. Nature Publishing Group; 2014;505: 361–6. 10.1038/nature12818 24352232

[5] Mullen SA, Carvill GL, Bellows S, Bayly M a, Trucks H, Lal D, et al Copy number variants are frequent in genetic generalized epilepsy with intellectual disability. Neurology. 2013;81: 1507–14. 10.1212/WNL.0b013e3182a95829 24068782 PMC3888172

[6] Jähn JA, von Spiczak S, Muhle H, Obermeier T, Franke A, Mefford HC, et al Iterative phenotyping of 15q11.2, 15q13.3 and 16p13.11 microdeletion carriers in pediatric epilepsies. Epilepsy Res. Elsevier B.V.; 2014;108: 109–116. 10.1016/j.eplepsyres.2013.10.001 24246141

[7] Chaste P, Sanders SJ, Mohan KN, Klei L, Song Y, Murtha MT, et al Modest impact on risk for autism spectrum disorder of rare copy number variants at 15q11.2, Specifically Breakpoints 1 to 2. Autism Res. 2014;7: 355–362. 10.1002/aur.1378 24821083 PMC6003409

[8] Burnside RD, Pasion R, Mikhail FM, Carroll AJ, Robin NH, Youngs EL, et al Microdeletion/microduplication of proximal 15q11.2 between BP1 and BP2: a susceptibility region for neurological dysfunction including developmental and language delay. Hum Genet. 2011;130: 517–28. 10.1007/s00439-011-0970-4 21359847 PMC6814187

[9] van der Zwaag B, Staal WG, Hochstenbach R, Poot M, Spierenburg HA, de Jonge M V, et al A co-segregating microduplication of chromosome 15q11.2 pinpoints two risk genes for autism spectrum disorder. Am J Med Genet B Neuropsychiatr Genet. 2010;153B: 960–6. 10.1002/ajmg.b.31055 20029941 PMC2933514

[10] Makris N, Goldstein JM, Kennedy D, Hodge SM, Caviness VS, Faraone S V, et al Decreased volume of left and total anterior insular lobule in schizophrenia. Schizophr Res. 2006;83: 155–71. 10.1016/j.schres.2005.11.020 16448806

[11] Arnone D, McIntosh AM, Tan GMY, Ebmeier KP. Meta-analysis of magnetic resonance imaging studies of the corpus callosum in schizophrenia. Schizophr Res. 2008;101: 124–132. 10.1016/j.schres.2008.01.005 18289833

[12] Bora E, Fornito A, Radua J, Walterfang M, Seal M, Wood SJ, et al Neuroanatomical abnormalities in schizophrenia: A multimodal voxelwise meta-analysis and meta-regression analysis. Schizophr Res. Elsevier B.V.; 2011;127: 46–57. 10.1016/j.schres.2010.12.020 21300524

[13] Guo W, Liu F, Liu J, Yu L, Zhang J, Zhang Z, et al Abnormal causal connectivity by structural deficits in first-episode, drug-naive schizophrenia at rest. Schizophr Bull. 2015;41: 57–65. 10.1093/schbul/sbu126 25170032 PMC4266300

[14] Liégeois F, Mayes A, Morgan A. Neural Correlates of Developmental Speech and Language Disorders: Evidence from Neuroimaging. Curr Dev Disord reports. 2014;1: 215–227. 10.1007/s40474-014-0019-1 PMC4104164 25057455

[15] Sliwinska MW, Khadilkar M, Campbell-Ratcliffe J, Quevenco F, Devlin JT. Early and sustained supramarginal gyrus contributions to phonological processing. Front Psychol. 2012;3: 161 10.3389/fpsyg.2012.00161 22654779 PMC3361019

[16] Murphy SM, Preble a M, Patel UK, O’Connell KL, Dias DP, Moritz M, et al GCP5 and GCP6: two new members of the human gamma-tubulin complex. Mol Biol Cell. 2001;12: 3340–3352. 11694571 10.1091/mbc.12.11.3340 PMC60259

[17] Izumi N, Fumoto K, Izumi S, Kikuchi A. GSK-3β regulates proper mitotic spindle formation in cooperation with a component of the γ-tubulin ring complex, GCP5. J Biol Chem. 2008;283: 12981–12991. 10.1074/jbc.M710282200 18316369

[18] Xiong Y, Oakley BR. In vivo analysis of the functions of gamma-tubulin-complex proteins. J Cell Sci. 2009;122: 4218–27. 10.1242/jcs.059196 19861490 PMC2776505

[19] Emamian ES, Hall D, Birnbaum MJ, Karayiorgou M, Gogos JA. Convergent evidence for impaired AKT1-GSK3beta signaling in schizophrenia. Nat Genet. 2004;36: 131–7. 10.1038/ng1296 14745448

[20] Freyberg Z, Ferrando SJ, Javitch JA. Roles of the Akt/GSK-3 and Wnt signaling pathways in schizophrenia and antipsychotic drug action. Am J Psychiatry. 2010;167: 388–96. 10.1176/appi.ajp.2009.08121873 19917593 PMC3245866

[21] Hur E-M, Zhou F-Q. GSK3 signalling in neural development. Nat Rev Neurosci. 2010;11: 539–51. 10.1038/nrn2870 20648061 PMC3533361

[22] Kobayashi K, Kuroda S, Fukata M, Nakamura T, Nagase T, Nomura N, et al p140Sra-1 (specifically Rac1-associated protein) is a novel specific target for Rac1 small GTPase. J Biol Chem. 1998;273: 291–5. Available: http://www.ncbi.nlm.nih.gov/pubmed/9417078 9417078 10.1074/jbc.273.1.291

[23] Schenck A, Bardoni B, Langmann C, Harden N, Mandel JL, Giangrande A. CYFIP/Sra-1 controls neuronal connectivity in Drosophila and links the Rac1 GTPase pathway to the fragile X protein. Neuron. 2003;38: 887–898. 10.1016/S0896-6273(03)00354-4 12818175

[24] Napoli I, Mercaldo V, Boyl PP, Eleuteri B, Zalfa F, De Rubeis S, et al The fragile X syndrome protein represses activity-dependent translation through CYFIP1, a new 4E-BP. Cell. 2008;134: 1042–54. 10.1016/j.cell.2008.07.031 18805096

[25] Schenck A, Bardoni B, Moro A, Bagni C, Mandel JL. A highly conserved protein family interacting with the fragile X mental retardation protein (FMRP) and displaying selective interactions with FMRP-related proteins FXR1P and FXR2P. Proc Natl Acad Sci U S A. 2001;98: 8844–9. 10.1073/pnas.151231598 11438699 PMC37523

[26] Bozdagi O, Sakurai T, Dorr N, Pilorge M, Takahashi N, Buxbaum JD. Haploinsufficiency of Cyfip1 produces fragile X-like phenotypes in mice. PLoS One. 2012;7: e42422 10.1371/journal.pone.0042422 22900020 PMC3416859

[27] Oguro-Ando A, Rosensweig C, Herman E, Nishimura Y, Werling D, Bill BR, et al Increased CYFIP1 dosage alters cellular and dendritic morphology and dysregulates mTOR. Mol Psychiatry. 2014; 10.1038/mp.2014.124 PMC4409498 25311365

[28] Yoon K-J, Nguyen HN, Ursini G, Zhang F, Kim N-S, Wen Z, et al Modeling a Genetic Risk for Schizophrenia in iPSCs and Mice Reveals Neural Stem Cell Deficits Associated with Adherens Junctions and Polarity. Cell Stem Cell. Elsevier Inc.; 2014;15: 79–91. 10.1016/j.stem.2014.05.003 PMC4237009 24996170

[29] De Rubeis S, Pasciuto E, Li KW, Fernández E, Di Marino D, Buzzi A, et al CYFIP1 coordinates mRNA translation and cytoskeleton remodeling to ensure proper dendritic spine formation. Neuron. 2013;79: 1169–82. 10.1016/j.neuron.2013.06.039 24050404 PMC3781321

[30] Wang J, Tao Y, Song F, Sun Y, Ott J, Saffen D. Common Regulatory Variants of CYFIP1 Contribute to Susceptibility for Autism Spectrum Disorder (ASD) and Classical Autism. Ann Hum Genet. 2015; 329–340. 10.1111/ahg.12121 26094621

[31] Jiang Y, Zhang Y, Zhang P, Sang T, Zhang F, Ji T, et al NIPA2 located in 15q11.2 is mutated in patients with childhood absence epilepsy. Hum Genet. 2012;131: 1217–24. 10.1007/s00439-012-1149-3 22367439

[32] Xie H, Zhang Y, Zhang P, Wang J, Wu Y, Wu X, et al Functional Study of NIPA2 Mutations Identified from the Patients with Childhood Absence Epilepsy. PLoS One. 2014;9: e109749 10.1371/journal.pone.0109749 25347071 PMC4209971

[33] Bittel DC, Kibiryeva N, Butler MG. Expression of 4 genes between chromosome 15 breakpoints 1 and 2 and behavioral outcomes in Prader-Willi syndrome. Pediatrics. 2006;118: e1276–83. 10.1542/peds.2006-0424 16982806 PMC5453799

[34] Tsang HTH, Edwards TL, Wang X, Connell JW, Davies RJ, Durrington HJ, et al The hereditary spastic paraplegia proteins NIPA1, spastin and spartin are inhibitors of mammalian BMP signalling. Hum Mol Genet. 2009;18: 3805–21. 10.1093/hmg/ddp324 19620182 PMC2748891

[35] Rainier S, Chai J-H, Tokarz D, Nicholls RD, Fink JK. NIPA1 gene mutations cause autosomal dominant hereditary spastic paraplegia (SPG6). Am J Hum Genet. 2003;73: 967–71. 10.1086/378817 14508710 PMC1180617

[36] Fisher SE, Scharff C. FOXP2 as a molecular window into speech and language. Trends Genet. 2009;25: 166–177. 10.1016/j.tig.2009.03.002 19304338

[37] Lai CS, Fisher SE, Hurst JA, Vargha-Khadem F, Monaco AP. A forkhead-domain gene is mutated in a severe speech and language disorder. Nature. 2001;413: 519–523. 10.1038/35097076 11586359

[38] Vernes SC, Newbury DF, Abrahams BS, Winchester L, Nicod J, Groszer M, et al A functional genetic link between distinct developmental language disorders. N Engl J Med. 2008;359: 2337–45. 10.1056/NEJMoa0802828 18987363 PMC2756409

[39] Barrett JC, Fry B, Maller J, Daly MJ. Haploview: Analysis and visualization of LD and haplotype maps. Bioinformatics. 2005;21: 263–265. 10.1093/bioinformatics/bth457 15297300

[40] Ross P, Hall L, Smirnov I, Haff L. High level multiplex genotyping by MALDI-TOF mass spectrometry. Nat Biotechnol. 1998;16: 1347–51. 10.1038/4328 9853617

[41] Franke B, Vasquez AA, Veltman JA, Brunner HG, Rijpkema M, Fernández G. Genetic variation in CACNA1C, a gene associated with bipolar disorder, influences brainstem rather than gray matter volume in healthy individuals. Biol Psychiatry. 2010;68: 586–8. 10.1016/j.biopsych.2010.05.037 20638048

[42] Guadalupe T, Zwiers MP, Teumer A, Wittfeld K, Vasquez AA, Hoogman M, et al Measurement and genetics of human subcortical and hippocampal asymmetries in large datasets. Hum Brain Mapp. 2014;35: 3277–89. 10.1002/hbm.22401 24827550 PMC6869341

[43] Guadalupe T, Zwiers MP, Wittfeld K, Teumer A, Vasquez AA, Hoogman M, et al Asymmetry within and around the human planum temporale is sexually dimorphic and influenced by genes involved in steroid hormone receptor activity. Cortex. 2014;2: 1–15. 10.1016/j.cortex.2014.07.015 25239853

[44] Reuter M, Schmansky NJ, Rosas HD, Fischl B. Within-subject template estimation for unbiased longitudinal image analysis. Neuroimage. 2012;61: 1402–18. 10.1016/j.neuroimage.2012.02.084 22430496 PMC3389460

[45] Dale AM, Fischl B, Sereno MI. Cortical surface-based analysis. I. Segmentation and surface reconstruction. Neuroimage. 1999;9: 179–94. 10.1006/nimg.1998.0395 9931268

[46] Desikan RS, Ségonne F, Fischl B, Quinn BT, Dickerson BC, Blacker D, et al An automated labeling system for subdividing the human cerebral cortex on MRI scans into gyral based regions of interest. Neuroimage. 2006;31: 968–80. 10.1016/j.neuroimage.2006.01.021 16530430

[47] R Core Team. R: A Language and Environment for Statistical Computing. Available: http://www.r-project.org/

[48] Purcell S, Neale B, Todd-Brown K, Thomas L, Ferreira M a R, Bender D, et al PLINK: a tool set for whole-genome association and population-based linkage analyses. Am J Hum Genet. 2007;81: 559–575. 10.1086/519795 17701901 PMC1950838

[49] Guadalupe T, Willems RM, Zwiers MP, Arias Vasquez A, Hoogman M, Hagoort P, et al Differences in cerebral cortical anatomy of left- and right-handers. Front Psychol. 2014;5: 261 10.3389/fpsyg.2014.00261 24734025 PMC3975119

[50] Ramasamy A, Trabzuni D, Guelfi S, Varghese V, Smith C, Walker R, et al Genetic variability in the regulation of gene expression in ten regions of the human brain. Nat Neurosci. Nature Publishing Group; 2014;17: 1418–1428. 10.1038/nn.3801 PMC4208299 25174004

[51] Mathelier A, Zhao X, Zhang AW, Parcy F, Worsley-Hunt R, Arenillas DJ, et al JASPAR 2014: An extensively expanded and updated open-access database of transcription factor binding profiles. Nucleic Acids Res. 2014;42: 142–147. 10.1093/nar/gkt997 PMC3965086 24194598

[52] Sandelin A, Höglund A, Lenhard B, Wasserman WW. Integrated analysis of yeast regulatory sequences for biologically linked clusters of genes. Funct Integr Genomics. 2003;3: 125–134. 10.1007/s10142-003-0086-6 12827523

[53] Purcell SM, Wray NR, Stone JL, Visscher PM, O’Donovan MC, Sullivan PF, et al Common polygenic variation contributes to risk of schizophrenia and bipolar disorder. Nature. 2009;460: 748–52. 10.1038/nature08185 19571811 PMC3912837

[54] Klei L, Sanders SJ, Murtha MT, Hus V, Lowe JK, Willsey a J, et al Common genetic variants, acting additively, are a major source of risk for autism. Mol Autism. Molecular Autism; 2012;3: 9 10.1186/2040-2392-3-9 23067556 PMC3579743

[55] Waltes R, Duketis E, Knapp M, Anney RJL, Huguet G, Schlitt S, et al Common variants in genes of the postsynaptic FMRP signalling pathway are risk factors for autism spectrum disorders. Hum Genet. 2014;133: 781–792. 10.1007/s00439-013-1416-y 24442360

[56] Chung L, Wang X, Zhu L, Towers A, Kim IH, Jiang Y-H. Parental origin impairment of synaptic functions and behaviors in cytoplasmic FMRP interacting protein 1 (Cyfip1) deficiency mice. Brain Res. Elsevier; 2015;1: 1–11. 10.1016/j.brainres.2015.10.015 PMC4744651 26474913

[57] Noble KG, Houston SM, Brito NH, Bartsch H, Kan E, Kuperman JM, et al Family income, parental education and brain structure in children and adolescents. Nat Neurosci. 2015;18: 773–778. 10.1038/nn.3983 25821911 PMC4414816

[58] Nonaka-Kinoshita M, Reillo I, Artegiani B, Martínez-Martínez MÁ, Nelson M, Borrell V, et al Regulation of cerebral cortex size and folding by expansion of basal progenitors. EMBO J. 2013;32: 1817–28. 10.1038/emboj.2013.96 23624932 PMC3926188

[59] Ecker C, Murphy D. Neuroimaging in autism—from basic science to translational research. Nat Rev Neurol. Nature Publishing Group; 2014;10: 82–91. 10.1038/nrneurol.2013.276 24419683

[60] Fisher SE, Vargha-Khadem F, Watkins KE, Monaco AP, Pembrey ME. Localisation of a gene implicated in a severe speech and language disorder. Nat Genet. 1998;18: 168–170. 10.1038/ng0298-168 9462748

[61] Graham SA, Fisher SE. Decoding the genetics of speech and language. Curr Opin Neurobiol. Elsevier Ltd; 2013;23: 43–51. 10.1016/j.conb.2012.11.006 23228431

[62] Liégeois F, Baldeweg T, Connelly A, Gadian DG, Mishkin M, Vargha-Khadem F. Language fMRI abnormalities associated with FOXP2 gene mutation. Nat Neurosci. 2003;6: 1230–1237. 10.1038/nn1138 14555953

[63] Linkersdörfer J, Lonnemann J, Lindberg S, Hasselhorn M, Fiebach CJ. Grey matter alterations co-localize with functional abnormalities in developmental dyslexia: an ALE meta-analysis. PLoS One. 2012;7: e43122 10.1371/journal.pone.0043122 22916214 PMC3423424

[64] Raschle NM, Chang M, Gaab N. Structural brain alterations associated with dyslexia predate reading onset. Neuroimage. 2011;57: 742–9. 10.1016/j.neuroimage.2010.09.055 20884362 PMC3499031

[65] Temple E. Brain mechanisms in normal and dyslexic readers. Curr Opin Neurobiol. 2002;12: 178–83. Available: http://www.ncbi.nlm.nih.gov/pubmed/12015234 12015234 10.1016/s0959-4388(02)00303-3

[66] Dufor O, Serniclaes W, Sprenger-Charolles L, Démonet J-F. Top-down processes during auditory phoneme categorization in dyslexia: a PET study. Neuroimage. 2007;34: 1692–707. 10.1016/j.neuroimage.2006.10.034 17196834

[67] Ruff S, Cardebat D, Marie N, Démonet JF. Enhanced response of the left frontal cortex to slowed down speech in dyslexia: an fMRI study. Neuroreport. 2002;13: 1285–9. Available: http://www.ncbi.nlm.nih.gov/pubmed/12151788 12151788 10.1097/00001756-200207190-00014

[68] Price CJ. A review and synthesis of the first 20years of PET and fMRI studies of heard speech, spoken language and reading. Neuroimage. Elsevier Inc.; 2012;62: 816–847. 10.1016/j.neuroimage.2012.04.062 PMC3398395 22584224

[69] Price CJ. The anatomy of language: a review of 100 fMRI studies published in 2009. Ann N Y Acad Sci. 2010;1191: 62–88. 10.1111/j.1749-6632.2010.05444.x 20392276

[70] Hickok G. Computational neuroanatomy of speech production. Nat Rev Neurosci. 2012;13: 135–45. 10.1038/nrn3158 22218206 PMC5367153

[71] Golfinopoulos E, Tourville JA, Guenther FH. The integration of large-scale neural network modeling and functional brain imaging in speech motor control. Neuroimage. 2010;52: 862–74. 10.1016/j.neuroimage.2009.10.023 19837177 PMC2891349

