# Supplementary figures and images for: A Common CYFIP1 Variant at the 15q11.2 Disease Locus Is Associated with Structural Variation at the Language-Related Left Supramarginal Gyrus
Source: PLoS One. 2016 Jun 28;11(6):e0158036. doi: 10.1371/journal.pone.0158036 (PMC4924813; doi:10.1371/journal.pone.0158036)

S1 Figure. Linkage disequilibrium (LD) structure of the breakpoint 1 to breakpoint 2 locus.

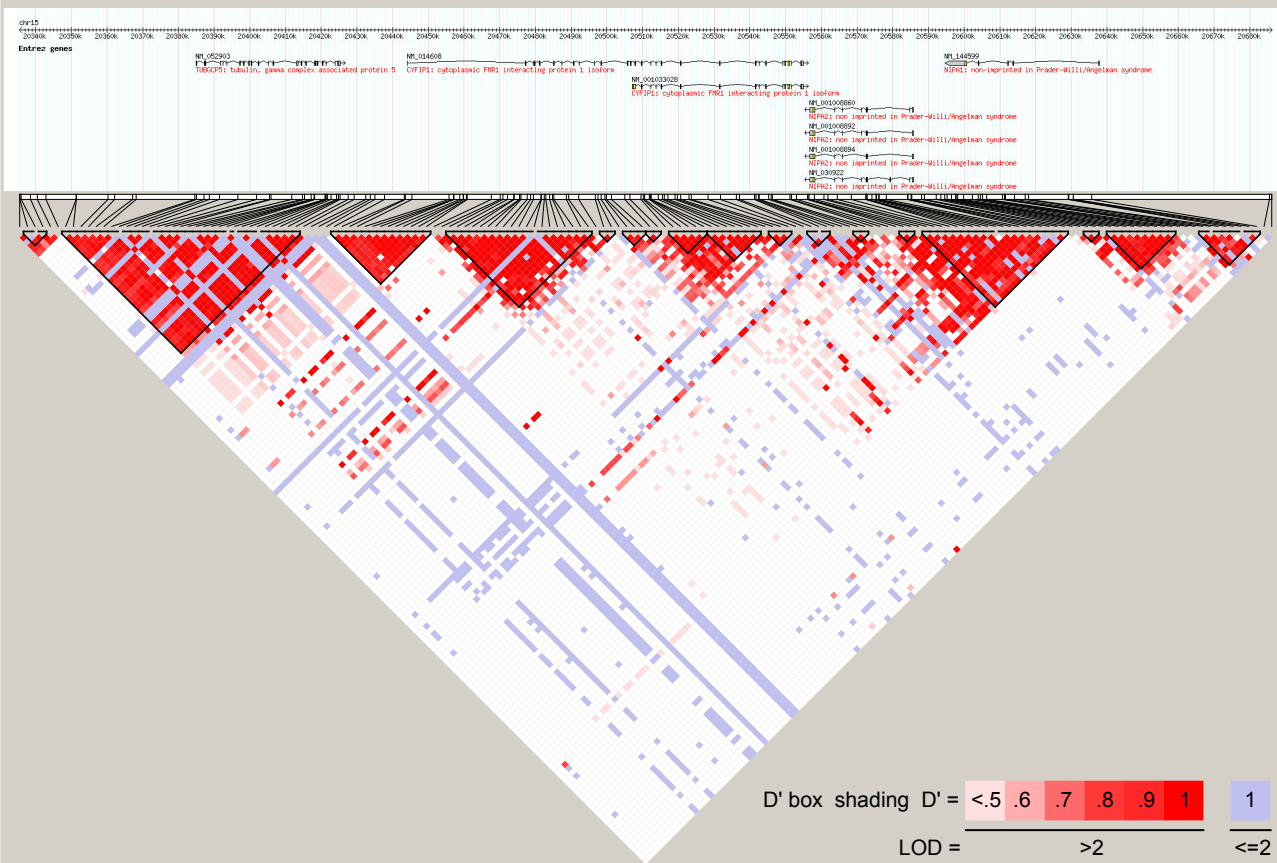

Supplement: S1 Fig — Haploview was used to generate a schematic representation of the interval and define LD structure. Genes within the interval are illustrated (TUBGCP5, CYFIP1, NIPA2, and NIPA1). Below this, SNPs are represented by short vertical lines. LD blocks appear as black triangles. (PDF) [file pone.0158036.s001.pdf]
